# Genomic epidemiology reveals multiple mechanisms of linezolid resistance in clinical enterococci in China

**DOI:** 10.1186/s12941-024-00689-0

**Published:** 2024-05-04

**Authors:** Ziran Wang, Danping Liu, Jingjia Zhang, Lingli Liu, Zeming Zhang, Chang Liu, Songnian Hu, Linhuan Wu, Zilong He, Hongli Sun

**Affiliations:** 1 Department of Clinical Laboratory, Peking Union Medical College Hospital, Peking Union Medical College, Chinese Academy of Medical Sciences, No.1 Shuaifuyuan Wangfujing, Dongcheng, Beijing, 100730 P.R. China; 2 School of Engineering Medicine, Beihang University, Rd37, xueyuan, Haidian, Beijing, 100191 P.R. China; 3 Key Laboratory of Big Data-Based Precision Medicine, Beihang University, Ministry of Industry and Information Technology of the People’s Republic of China, Beijing, China; 4 Key Laboratory of Biomechanics and Mechanobiology, Beihang University, Ministry of Education, Beijing, China; 5 Department of Clinical Laboratory, Beijing Huaxin Hospital, The First Hospital of Tsinghua University, Beijing, China; 6 State Key Laboratory of Microbial Resources, Institute of Microbiology, Chinese Academy of Sciences, Beijing, China; 7 Microbial Resource and Big Data Center, Institute of Microbiology, Chinese Academy of Sciences, Beijing, China

**Keywords:** *Enterococcus faecalis*, *Enterococcus faecium*, Linezolid, *optrA*, Whole-genome sequencing

## Abstract

**Background:**

Infections caused by linezolid-resistant enterococci (LRE) are clinically difficult to treat and threaten patient health. However, there is a lack of studies on long time-span LRE strains in China. For this reason, our study comprehensively revealed the resistance mechanisms of LRE strains collected in a Chinese tertiary care hospital from 2011 to 2022.

**Methods:**

Enterococcal strains were screened and verified after retrospective analysis of microbial data. Subsequently, 65 LRE strains (61 *Enterococcus faecalis* and 4 *Enterococcus faecium*, MIC ≥ 8 µg/ml), 1 linezolid-intermediate *Enterococcus faecium* (MIC = 4 µg/ml) and 1 linezolid-susceptible *Enterococcus faecium* (MIC = 1.5 µg/ml) were submitted for whole-genome sequencing (WGS) analysis and bioinformatics analysis.

**Results:**

The *optrA* gene was found to be the most common linezolid resistance mechanism in our study. We identified the wild-type OptrA and various OptrA variants in 98.5% of LRE strains (61 *Enterococcus faecalis* and 3 *Enterococcus faecium*). We also found one linezolid-resistant *Enterococcus faecium* strain carried both *optrA* and *cfr*(D) gene, while one linezolid-resistant *Enterococcus faecium* only harbored the *poxtA* gene. Most *optrA* genes (55/64) were located on plasmids, with *impB*-*fexA*-*optrA*, *impB*-*fexA*-*optrA*-*erm*(A), *fexA*-*optrA*-*erm*(A), and *fexA*-*optrA* segments. A minority of *optrA* genes (9/64) were found on chromosomes with the Tn*6674*-like platform. Besides, other possible linezolid resistance-associated mechanisms (mutations in the *rplC* and *rplD* genes) were also found in 26 enterococcal strains.

**Conclusions:**

Our study suggested that multiple mechanisms of linezolid resistance exist among clinical LRE strains in China.

**Supplementary Information:**

The online version contains supplementary material available at 10.1186/s12941-024-00689-0.

## Introduction

*Enterococcus* species (enterococci) are Gram-positive bacteria widely spread in the environment and hospital. They are regarded as opportunistic pathogens, which can colonize the gut of humans or animals as well as can lead to healthcare-associated infections. Enterococci may be responsible for various infections, including bacteremia, urinary tract infection, endocarditis, surgical site infections, and root canal failure [1]. Concerningly, enterococci show intrinsic resistance to commonly used antibiotics, including cephalosporins, aminoglycosides, clindamycin, and trimethoprim-sulfamethoxazole [2]. Additionally, the emergence and rapid expansion of vancomycin-resistant enterococci (VRE) strains has narrowed the therapeutic options [3]. Even worse, the property of enterococci to acquire resistance genes through plasmids or other genetic elements makes infections difficult to control [4].

Linezolid is the first oxazolidinone antibiotic approved by the U.S. Food and Drug Administration (FDA) for clinical use. Linezolid has demonstrated clinical benefits in treating severe Gram-positive bacterial infections caused by VRE, multidrug-resistant *Streptococcus pneumoniae*, and the challenging methicillin-resistant *Staphylococcus aureus* (MRSA) [5]. Unfortunately, in the last few years, reports of linezolid-resistant enterococci (LRE) have begun to appear and increase worldwide [6–10]. From 2000 to 2016, the bloodstream infections caused by linezolid-resistant *Enterococcus faecium * (*E. faecium*) and *Enterococcus faecalis* (*E. faecalis*) increased globally from 0.8% and 0.3% to 3% and 2%, respectively [11]. Although the detection rate of LRE is not high at present, the threat posed by its spread may make enterococcal infections uncontrollable.

Several specific mechanisms have been reported to be associated with LRE. Since linezolid inhibits polypeptide synthesis and elongation by binding to 23S rRNA, mutations in 23S rRNA can reduce the susceptibility to linezolid in enterococci [12]. Among these, G2505A or G2576U substitutions in domain V on 23S rRNA were more common [13]. Alternatively, mutations in the ribosomal proteins L3 (*rplC*) and L4 (*rplD*) could also confer resistance to linezolid [14]. The *cfr* gene was initially identified on the multi-resistance plasmid from a bovine strain of *Staphylococcus sciuri*, which encodes the 23S rRNA methyltransferase that confers multi-resistance against phenicols, lincosamides, oxazolidinones, pleuromutilins, and streptogramin A (PhLOPS_A_ phenotype) [15]. Furthermore, *cfr*(B) and *cfr*(D), as variants of *cfr*, have also been observed as mobile genetic elements in enterococci [16, 17]. In addition, it has been reported that the *optrA* gene encodes the ATP-binding cassette (ABC) protein that may confer transferable resistance to oxazolidinones and phenicols through a ribosomal protection mechanism [18]. The *optrA* gene was first identified in China in *E. faecalis* and *E. faecium* strains of human and animal origin but was subsequently detected in enterococci from more than 20 countries [10, 19, 20]. Another novel phenicol-oxazolidinone-tetracycline resistance gene, *poxtA*, was characterized in the chromosome of a MRSA of clinical origin [21]. The *poxtA* gene has been reported to be detected in enterococci isolated from humans, animals, and environmental sources [18]. In terms of the genomic context, the conserved structure constituted by the flanking IS*1216* of the *poxtA* gene is associated with its mobility [22].

There are relatively few reports on LRE in China. These studies have collectively pointed out that the co-existence of multiple resistance mechanisms in LRE, which may be a worrisome issue [23–26]. Moreover, the clonal relatedness and genetic context of clinical LRE strains in China have not been fully elucidated. Existing studies mostly used polymerase chain reaction (PCR) or Sanger sequencing to detect the mechanism of linezolid resistance in enterococci [27, 28]. However, these studies may need more precise identification for the resistance mechanism. On this basis, this study retrospectively analyzed the prevalence of LRE isolated from our healthcare institution from 2011 to 2022 and used whole-genome sequencing (WGS) to explore these isolated strains’ clonal correlations and resistance mechanisms.

## Materials and methods

### Bacterial strains and antimicrobial susceptibility testing (AST)

The overall design of this study is displayed in Figure S1. A total of 5779 enterococci strains were isolated at Peking Union Medical College Hospital, a tertiary care hospital in Beijing, from 2011 to 2022. These strains were continuously isolated from non-repeat clinical patients and were all pathogenic or conditioned pathogens. The species of the strains were identified using the matrix-assisted laser desorption/ionization time of flight mass spectrometry (MALDI-TOF MS; bioMérieux, Lyons, France). In clinical practice at our institution, the Kirby-Bauer disk diffusion method or broth microdilution method was routinely used to determine the susceptibility of enterococci to linezolid. The linezolid susceptibility testing results of all enterococci were retrospectively analyzed by the Laboratory Information System (LIS) and 65 LRE strains were screened. Furthermore, all screened LRE strains were revalidated using the E-test method. Therefore, all minimum inhibitory concentration (MIC) values of LRE strains against linezolid in this study were derived from the E-test method. Antimicrobial susceptibility profiles of 65 LRE strains were also subsequently determined using the broth microdilution method. The MIC values of 65 LRE strains against various antibiotics, including PEN (Penicillin), AMP (Ampicillin), TGC (Tigecycline), ERY (Erythromycin), TEC (Teicoplanin), VAN (Vancomycin), LEV (Levofloxacin), FOS (Fosfomycin), NIT (Nitrofurantoin), TCY (Tetracycline), DAP (Daptomycin), CHL (Chloramphenicol), RIF (Rifampicin) and MI (Minocycline), were determined and interpreted according to the Clinical and Laboratory Standards Institute (CLSI) and European Committee on Antimicrobial Susceptibility Testing (EUCAST) guidelines. *E. faecalis* ATCC 29212 was used as the quality control strain. Patient characteristics and disease information corresponding to the strains were obtained from the hospital information system (HIS). This study was conducted in accordance with the guidelines of the Helsinki Declaration and was approved by Ethics Committee of Peking Union Medical College Hospital (Approval No. I-23PJ1724).

### Detection of linezolid resistance-associated genes using PCR

Two non-LRE *E. faecium* strains (L1 and L49) were submitted to detect whether linezolid resistance genes [*optrA*, *cfr*, *cfr(B)*, *cfr(D)* and *poxtA*] were present by using PCR. The sequences of the primers were derived from a previous literature [9]. Since the Tm values of all primers are close to 59 °C, we chose the same annealing condition for the different PCR reactions targeting various resistance genes. The detailed amplification conditions were: pre-denaturation (94 °C for 30s), 30 amplification cycles (94 °C for 30s, 56 °C for 30s, 72 °C for 30s), extension (72 °C for 5 min).

### DNA extraction, library construction, and whole-genome sequencing

65 LRE strains and 2 non-LRE strains (L1 and L49) were submitted for whole genome sequencing (WGS) analysis. Genomic DNA was extracted from pure cultures using a commercial kit (QIAGEN, United States) and quantified by Qubit 4.0 (USA Invitrogen ABI). Whole-genome shotgun DNA was used for library preparation using either PCR-based protocol (MGIEasy FS DNA Library Prep Set, containing PCR-amplification steps after second bead purification). One portion of the genomic DNA was fragmented to about 5–10 kbp. Sequencing libraries were constructed using the MGI Easy Universal DNA Library Prep Set. All libraries were then sequenced on the MGISEQ-2000 platform with the PE150 model.

### Data preprocessing, genome assembly, gene prediction, and type determination

Low quality, PCR duplication and adapter sequence were removed from raw data by Trim Galore (version 0.6.7, https://github.com/FelixKrueger/TrimGalore). Genome assembly and gene prediction of each strain were performed by SPAdes [29] (version 3.15.4) and Prokka [30] (version 1.14.6), respectively. Taxonomy classification of each strain was performed by Kraken [31] (version 2.1.2) and Bracken [32] (version 2.6.1). The completeness and contamination of final assemblies were evaluated using CheckM [33] (version 1.2.2). Genome statistics were evaluated by QUAST [34] (version 5.2.0). Multi-locus sequence typing (MLST) of each strain was determined by using mlst (version 2.23.0, https://github.com/tseemann/mlst) based on the PubMLST website (https://pubmlst.org/).

### Other bioinformatics analyses

The whole-genome phylogenetic tree of strains was built using PhyloPhlan [35] (version 3.0.67) and RaxML [36] (version 8.2.12). PhyloPhlan used the parameters “--diversity low --fast -d phylophlan” and RaxML used the parameters “-f a -x 12345 -p 12345 -# 1000 -m PROTGAMMAAUTO”. LRE-Finder [13] (version 1.0.0) was applied to detect linezolid resistance genes [*optrA*, *cfr*, *cfr*(B), and *poxtA*] and common mutations in the V domain of the 23S rRNA (G2576U or G2505A) in enterococci. The Resistance Gene Identifier (version 4.0.3) was used to predict other linezolid resistance genes [*cfr*(D)] based on the reference data from the Comprehensive Antibiotic Resistance Database (CARD) [37] (version 3.2.6). All identified linezolid resistance genes were verified through the blastp (DIAMOND [38], version 2.0.15.153) alignment against the non-redundant protein sequences database (NR) (screening parameters: identity ≥ 99%, e-value < 1e-10). The location of linezolid resistance genes was predicted by Plasmer [39] (version 0.1). The predicted plasmid sequences (greater than 10 kp in length) containing linezolid resistance genes were annotated through the online BLAST software (https://blast.ncbi.nlm.nih.gov/Blast.cgi) with default parameters. The multiple sequence alignments among *optrA* genes were performed by MAFFT [40] (version 7.520) with parameters: ‘‘--localpair --maxiterate 1000’’. The phylogenetic tree of OptrA proteins was constructed by RaxML [36] (version 8.2.12) with default parameters and chose the wild-type OptrA sequence (NG_048023.1) as the outgroup. Phylogenetic trees were visualized using ggtree [41] (version 3.2.1). The protein variants of *optrA* genes were identified by alignments with NG_048023.1 as a reference. The PROVEAN tool [42] (http://provean.jcvi.org/) was used to predict amino acid mutation effects. To identify the genetic variation in the *rplC* (ribosomal protein L3) and *rplD* (ribosomal protein L4) genes, the multiple sequence alignments of those genes were also performed by MAFFT [40] (version 7.520) with CP003583.1 and CP008816.1 as references. In gene structure analysis, IslandViewer 4 [43] (http://www.pathogenomics.sfu.ca/islandviewer/) was applied in the prediction of genomic islands. The ISfinder [44] platform (http://www-is.biotoul.fr) was used for annotating insertion sequences (IS). The comparison of gene clusters was visualized by Easyfig [45] (version 2.2.5).

## Results

### Prevalence, clinical characteristics, antimicrobial susceptibility, and genomic data statistics of LRE

From 2011 to 2022, a total of 5779 enterococci isolates were isolated. Of these, *E. faecalis* (3514/5779, 60.8%) and *E. faecium* (2114/5779, 36.6%) accounted for the majority, while *Enterococcus avium* (69/5779, 1.2%) and other enterococci (82/5779, 1.4%) represented a relatively small proportion. Regarding linezolid resistance, a total of 61 *E. faecalis* and 4 *E. faecium* strains were recognized as LRE (MIC ≥ 8 µg/ml). The overall LRE prevalence rate is 1.1% (65/5779). Specifically, the prevalence of LRE in *E. faecalis* and *E. faecium* was 1.7% (61/3514) and 0.2% (4/2114), respectively. The MIC range of these LRE strains against linezolid was 8–48 µg/ml. Most of them were isolated in 2020 and 2021. The clinical characteristics of the patients who isolated these strains were presented in Table S1. Most of these patients were female and ranged from newborn to 89 years old. Patients were usually admitted to Surgery (SUR), Pediatrics (PED), or General Medicine (MED) departments. There was a wide range of sample types, including drainage fluid, wound secretions, swabs, and peripheral blood. In terms of patients’ conditions, it is of note that patients with malignant tumors, infections, and preterm deliveries carried LRE in this study. The antimicrobial susceptibility results of 65 LRE strains are displayed in Table 1. All LRE strains were susceptible to TGC, FOS, and NIT, whereas all were resistant to TCY. Additionally, most of the strains were also susceptible to TEC, VAN, PEN, and AMP. For CHL, ERY, and LEV, most strains exhibit resistance. Moreover, 58.5%, 52.3% and 46.2% of LRE strains exhibited intermediate to MI, DAP, and RIF, respectively.

**Table 1:** The antimicrobial susceptibility testing results of 65 LRE strains

Antibiotic	S%	I%	R%
PEN	59/65 (90.8%)	0/65 (0%)	6/65 (9.2%)
AMP	59/65 (90.8%)	0/65 (0%)	6/65 (9.2%)
TGC	65/65 (100%)	0/65 (0%)	0/65 (0%)
ERY	1/65 (1.5%)	4/65 (6.2%)	60/65 (92.3%)
TEC	64/65 (98.5%)	0/65 (0%)	1/65 (1.5%)
VAN	64/65 (98.5%)	0/65 (0%)	1/65 (1.5%)
LEV	9/65 (13.8%)	1/65 (1.5%)	55/65 (84.6%)
FOS	65/65(100%)	0/65 (0%)	0/65 (0%)
NIT	65/65(100%)	0/65 (0%)	0/65 (0%)
TCY	0/65 (0%)	0/65 (0%)	65/65 (100%)
DAP	26/65 (40.0%)	34/65 (52.3%)	5/65 (7.7%)
CHL	0/65 (0%)	4/65 (6.2%)	61/65 (93.8%)
RIF	31/65 (47.7%)	30/65 (46.2%)	4/65 (6.2%)
MI	9/65 (13.8%)	38/65 (58.5%)	18/65 (27.7%)

Note: S, susceptible; I, intermediate; R, resistant; PEN, Penicillin; AMP, Ampicillin; TGC, Tigecycline; ERY, Erythromycin; TEC, Teicoplanin; VAN, Vancomycin; LEV, Levofloxacin; FOS, Fosfomycin; NIT, Nitrofurantoin; TCY, Tetracycline; DAP, Daptomycin; CHL, Chloramphenicol; RIF, Rifampicin; MI, Minocycline

Whole-genome sequencing was performed on 65 LRE and 2 non-LRE strains. Non-LRE strains contained *E. faecium* strain L1 (intermediate, MIC = 4 µg/ml) and *E. faecium* strain L49 (susceptible, MIC = 1.5 µg/ml). The genomes of 67 enterococcal strains had an average genome size of 2.96 Mbp (range from 2.69 to 3.28 Mb), an average scaffold number of 96 (range from 31 to 495), and an average N50 of 0.46 Mb (range from 35.7 kb − 1.47 Mb). Detailed quality assessment results can be found in Table S2. Based on the PubMLST website (https://pubmlst.org/), 67 strains were classified into 15 sequence types (STs). ST16 was most prevalent among 61 *E. faecalis* strains (52.5%). ST547, ST555, and ST1693 were identified among *E. faecium* strains. Besides, STs of 4 *E. faecalis* and 3 *E. faecium* strains were not identified. The detailed distribution of sequence types is shown in Figure S2.

### Phylogenetic tree of enterococci

**Fig. 1:**
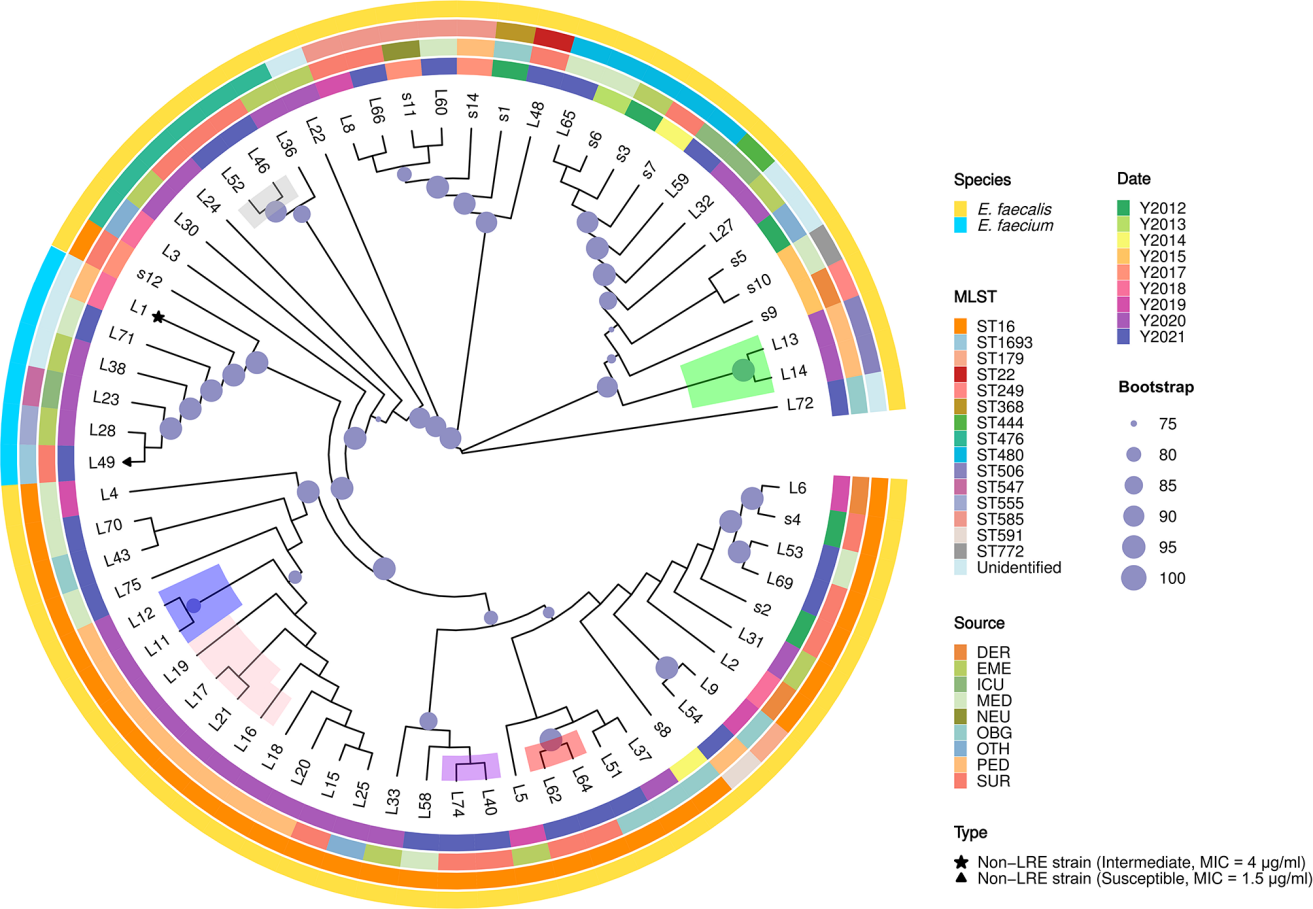
Phylogenetic tree of 67 enterococcal strains. Different circles represent different types of annotations. From the outermost to the innermost layer, different colors represent the species information, sequence type, department, and isolation time of the strain. Nodes with the same color indicate that the corresponding strain was isolated from the same individual at different times. Pentagrams and triangles represent different non-LRE strains, the rest of strains are all linezolid-resistant. Bootstrap values of 1000 repetitions of sampling are labelled on the evolutionary tree nodes

We constructed a phylogenetic tree based on whole genomes to determine the evolutionary relationships among 65 LRE and 2 non-LRE strains (Fig. 1). Overall, strains with the same ST type tended to cluster in the same clade, such as the ST16, ST476, ST480, and ST585 of *E. faecalis*. In addition, we noted that the evolutionary relationships of strains did not correlate significantly with the time and department of isolation. For example, *E. faecalis* strain s4 (isolated from Surgery in 2012) and *E. faecalis* strain L6 (isolated from Dermatology in 2019) were in the same clade, and *E. faecalis* strain s1 (isolated from Obstetrics and Gynecology in 2012) was closely evolutionarily related to *E. faecalis* strain L48 (isolated from Surgery in 2021).

### Identifying the mechanism of linezolid resistance

Linezolid resistance-associated mutations in the 23S rRNA, *rplC*, and *rplD* genes, as well as the carriage of linezolid resistance genes [*optrA*, *cfr*, *cfr*(B), *cfr*(D), and *poxtA*], have been reported to be the main mechanisms of linezolid resistance in enterococci [16, 46]. We comprehensively identified these mechanisms in 65 LRE and 2 non-LRE strains based on the next-generation sequencing (NGS) technique. The results revealed that carriage of the *optrA* gene was the most common linezolid resistance mechanism (Table S3). Collectively, the *optrA* genes were detected in 100% of linezolid-resistant *E. faecalis* strains (*n* = 61, MIC: 8–48 µg/ml) and 75% of linezolid-resistant *E. faecium* strains (*n* = 3, MIC: 16–24 µg/ml). Notably, no other resistance genes associated with linezolid resistance were detected in *E. faecalis* strains. Among four linezolid-resistant *E. faecium* strains, two only carried the *optrA* gene (MIC = 16 µg/ml and MIC = 24 µg/ml, respectively), one had both the *optrA* gene and the *cfr*(D) gene (MIC = 16 µg/ml), and one only carried the *poxtA* gene (MIC = 32 µg/ml). No resistance genes associated with linezolid resistance were detected in one linezolid-intermediate *E. faecium* strain (L1, MIC = 4 µg/ml) and one linezolid-susceptible *E. faecium* (L49, MIC = 1.5 µg/ml) strain. To further investigate whether there were linezolid resistance-associated genes in these two non-LRE strains, we validated the WGS results using the PCR assay. The PCR results showed that the *optrA*, *cfr*, *cfr(B)*, *cfr(D)* and *poxtA* genes were not detected in non-LRE strains. Based on the pre-developed machine learning model [39], we also predicted the plasmids in the assembled genomes of enterococcal strains to infer the location of the resistance genes *optrA*, *cfr*(D), and *poxtA*. The results showed that *cfr*(D), *poxtA*, and 85.9% (55/64) of the *optrA* genes were located on plasmids while only 14.1% (9/64) of the *optrA* genes were located on the chromosomes. Detailed location information can be found in Table S3.

In addition to the resistance genes, we detected several mutations in *rplC* and *rplD* genes, but none of these mutations resulted in alterations in the corresponding amino acid sequences. Overall, 7.5% (5/67) and 35.8% (24/67) of enterococcal strains contained mutations in *rplC* and *rplD* genes, respectively. The mutations on the *rplC* gene were mainly C369T (1 strain), T600C (3 strains), and C606T (1 strain). Moreover, A75T, T93G, T495C, C537A, T585G and T600C mutations were identified in the linezolid-intermediate *E. faecium* strain L1 (MIC = 4 µg/ml). The mutations on the *rplD* gene were predominantly the C348T type (20 strains), which has been reported previously [24]. Moreover, the common mutations (G2576U, G2505A) on the 23S rRNA associated with linezolid resistance were not detected in any of the 67 enterococcal strains. The identification results of linezolid resistance mechanisms in enterococci are detailed in Table S3.

### Bioinformatic analysis of *optrA* genes

**Fig. 2:**
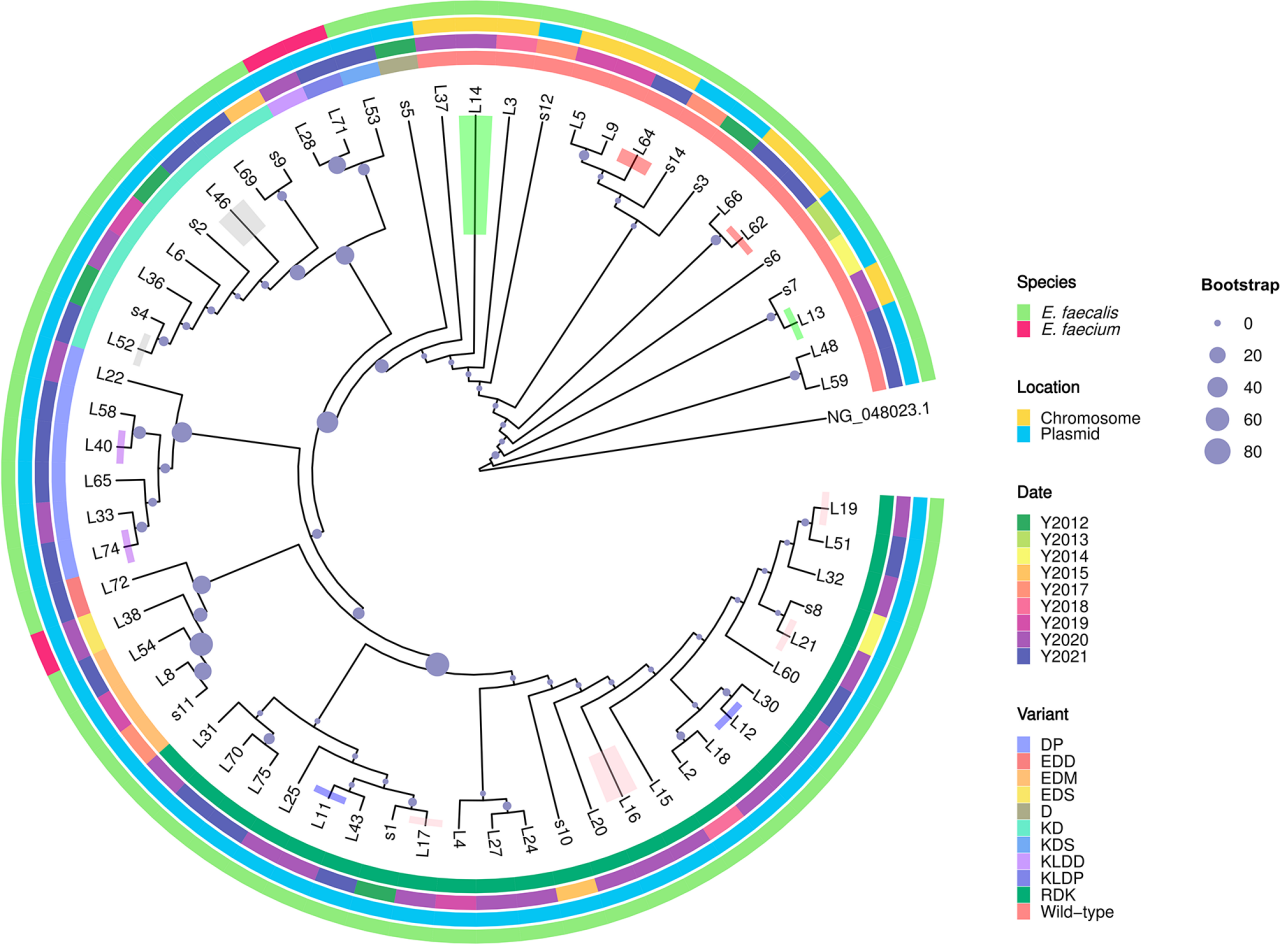
Phylogenetic tree of OptrA proteins. The first appeared OptrA protein (NG_048023.1) represents an outgroup of the evolutionary tree. Different circles represent different types of annotations. From the outermost to the innermost layer: the species information of the corresponding strain, the location of the *optrA* gene, the isolation time of the corresponding strain, and the type of the OptrA variant. Branches with the same color indicate that the corresponding strain was isolated from the same individual at different times. Bootstrap values for 1000 repetitions of sampling are labelled on the evolutionary tree nodes

Given the *optrA* gene was found to be the primary resistance mechanism to linezolid of enterococci in this study, we conducted further studies on these *optrA* genes with different sources. By comparing the OptrA proteins from 64 enterococcal strains with reference sequence in the database (NCBI Reference Sequence: NG_048023.1), we constructed a phylogenetic tree of OptrA proteins (Fig. 2). The phylogenetic tree demonstrated the topology between the wild-type OptrA and different variants. Overall, we identified the wild-type OptrA (16 strains) and ten OptrA variants (48 strains). Of all OptrA variants, the D variant was closest to the wild-type OptrA in terms of evolutionary relationship. In contrast, the RDK variant was furthest away from the wild-type OptrA in the phylogenetic tree. Through alignments, we found a total of four unreported *optrA* variants, namely the D and KDS variants in *E. faecalis* (both MIC = 8 µg/ml) and the KLDD and EDS variants in *E. faecalis* (MIC = 16 and MIC = 24 µg/ml, respectively). More information can be found in Table S3. To further explore the potential effects of amino acid mutations, we performed the mutation effects prediction analysis (Table S4). Of all amino acid substitutions on OptrA proteins, Y176D and G393D were predicted to be deleterious to protein function, whereas other substitutions were neutral. Except for the D variant (G394D), the other nine OptrA variants had the Y176D amino acid mutation, covering 44 *E. faecalis* and 3 *E. faecium* strains. The G393D mutation was only present in one *E. faecalis* strain (EDD variant) and one *E. faecalis* strain (KLDD variant). Additionally, we noted that the type of OptrA variants did not correlate with their location on the genome. Specifically, nine *optrA* genes on the chromosome were wild-type, while the other genes of the wild-type OptrA and OptrA variants were on the plasmid. After annotating the plasmids, we found that different genes of OptrA variants were present on different plasmids (Table S3). The newly discovered D, KDS, and KLDD variants were on plasmids pAR_0780, pEFs17-1, and pDY28-*optrA*, respectively, while 5 DP variants were located on the plasmid p661-b. Furthermore, the isolation time of strains with the same OptrA variant type varied. RDK variant, the most common OptrA variant in this study (25 strains), originated from strains isolated in 2012 and 2014 and those isolated in 2020 and 2021.

**Fig. 3:**
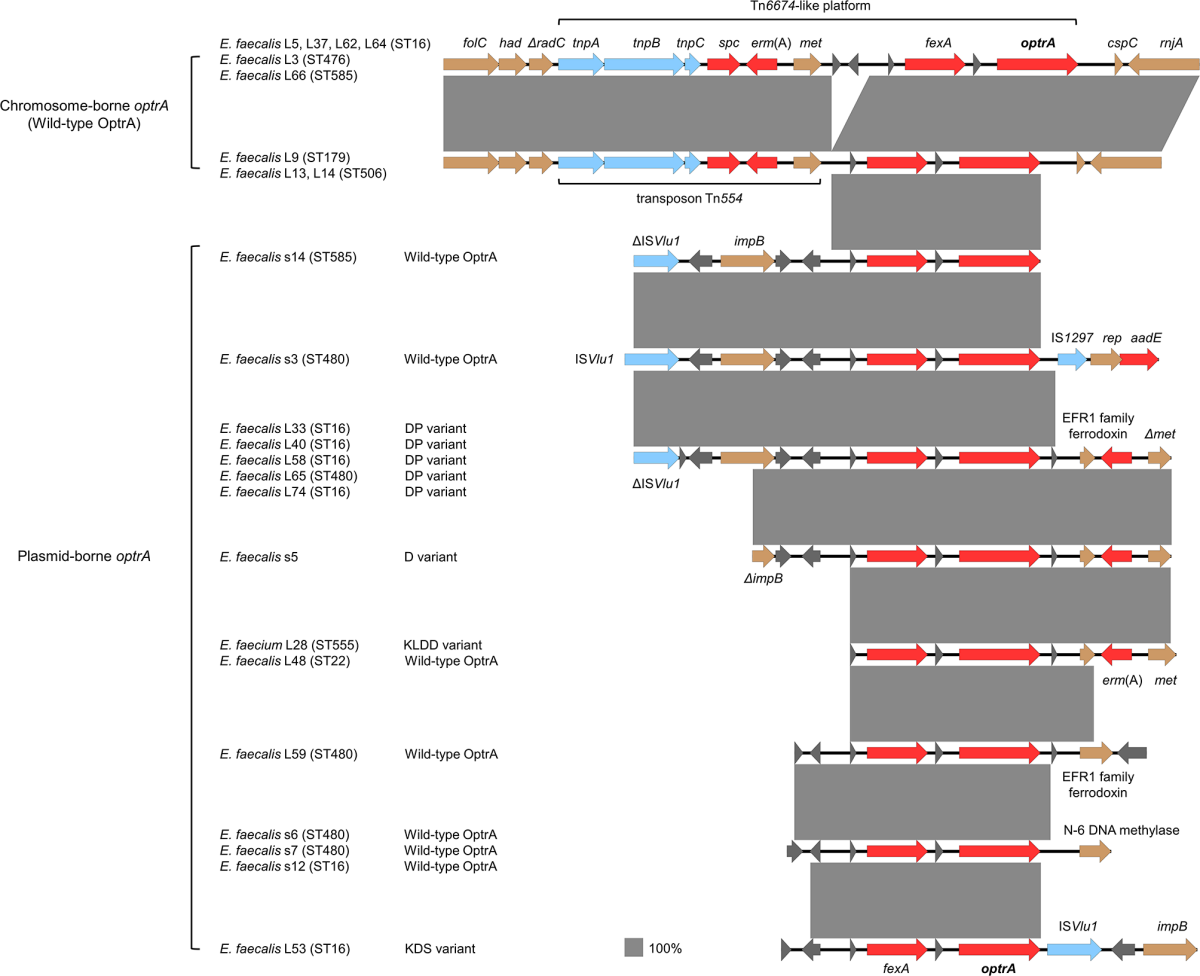
Genomic upstream and downstream structures of the *optrA* gene. Red indicates resistance genes, blue indicates mobile genetic elements, dark grey indicates genes encoding hypothetical proteins, and brown indicates other genes

Since the *optrA* gene was the most common linezolid resistance gene in this study, we analyzed the genetic context of all chromosomal *optrA* (*n* = 9) and some *optrA* plasmids (greater than 10 kp in length) (*n* = 15) (Fig. 3). We found that the upstream and downstream structures of chromosomal *optrA* genes were significantly different from those present on plasmids. On chromosomal sequences from 9 strains, clusters of genes carrying *optrA* (from *folC* to *rnjA*) were predicted to be genomic islands, indicating that this region might be associated with horizontal gene transfer. In this *optrA*-carrying region, we identified the Tn*6674*-like platform, consisting of genes *tnpA*, *tnpB*, and *tnpC* (encoding proteins involved in the transposition of transposon Tn*554*), *spc* (resistance to spectinomycin), *erm*(A) (resistance to macrolides, lincosamides, and streptogramin B antibiotics), *met* (encoding methyltransferase), *fexA* (resistance to phenicols), and *optrA* (resistance to oxazolidinones and phenicols). The structures of chromosomal *optrA* genes were basically the same, whereas the genetic context of *optrA* plasmids was diverse. The *impB*-*fexA*-*optrA* plasmid segment was identified in 2 strains of wild-type OptrA (*E. faecalis* s3 and s14), insertion sequences IS*Vlu1* and IS*1297* may affect the transfer and expression of this fragment. The *impB*-*fexA*-*optrA*-*erm*(A) arrangement was identified in 5 strains of DP variant (*E. faecalis* L33, L40, L58, L65, and L74). We observed the *fexA*-*optrA*-*erm*(A) arrangement in 3 strains, involving the D variant (*E. faecalis* s5), KLDD variant (*E. faecium* L28), and wild-type OptrA (*E. faecalis* L48). In 4 strains of wild-type OptrA (*E. faecalis* L59, s6, s7, and s12) and 1 strain of KDS variant (*E. faecalis* L53), we also identified the *fexA*-*optrA* plasmid segment.

## Discussion

Linezolid has been an essential antibiotic for the treatment of Gram-positive bacterial infections in clinical settings. However, infections caused by LRE have become global public health challenges. A meta-analysis estimated the global prevalence of linezolid-resistant *E. faecalis* and linezolid-resistant *E. faecium* to be 2.2% and 1.1%, respectively [47]. A 6-year surveillance from a teaching hospital in China showed that 3.93% (31/789) and 0.24% (2/834) of *E. faecalis* and *E. faecium*, respectively, exhibited resistance to linezolid [23]. In this study, we retrospectively analyzed microbial data from 2011 to 2022 and revealed that 1.7% (61/3514) and 0.2% (4/2114) of *E. faecalis* and *E. faecium* were resistant to linezolid, respectively. Although there may be variations in the prevalence of LRE, both our study and above studies implied that *E. faecalis* could be more likely to be resistant to linezolid than *E. faecium*. In terms of antimicrobial susceptibility testing results, most of the LRE strains isolated at our institution were susceptible to tigecycline, fosfomycin, nitrofurantoin, teicoplanin, vancomycin, penicillin, and ampicillin while resistant to tetracycline, chloramphenicol, erythromycin, and levofloxacin. Analogous to our findings, Li et al. observed that all LRE strains isolated at their institution were resistant to tetracycline, kanamycin, erythromycin, and ciprofloxacin but all were susceptible to vancomycin and teicoplanin [9]. In another report, all 22 LRE strains were confirmed to be susceptible to vancomycin, ampicillin, teicoplanin, and penicillin [23]. Taken together, vancomycin appears to be an effective option for the treatment of LRE, but intensive surveillance is necessary to prevent the emergence and rapid expansion of enterococci which resistant to both vancomycin and linezolid.

In this study, carriage of the *optrA* gene was the primary resistance mechanism of LRE strains (98.5%, 64/65) at our institution. However, this phenomenon was different from other researches. Zhang et al. analyzed 33 LRE strains collected from a teaching hospital in Wenzhou, China, and found that 54.6% carried both *cfr* and *optrA* genes [23]. Egan et al. studied 154 strains of LRE strains collected from 14 hospitals in Ireland and found that most of the strains had the G2576U 23S rRNA mutation or carried the *poxtA* gene, and only 9.7% of the strains carried the *optrA* gene [48]. Moure et al. studied different hospitals from Spain on 97 LRE strains and found that most strains carried the *optrA* gene or G2576U mutation [49]. Sassi et al. analyzed nine LRE strains collected from French hospitals between 2006 and 2016 and found that eight carried the *optrA* gene [8]. Overall, there may be variations in the primary resistance mechanism of LRE across different regions, which could be associated with discrepancies in healthcare conditions as well as antibiotic application strategies in various regions. Two other resistance genes [*cfr*(D) and *poxtA*] associated with linezolid resistance were identified in two *E. faecium* strains respectively in this study. Interestingly, one *E. faecium* strain harboring the *cfr*(D) gene also carried the *optrA* gene. This co-occurrence pattern was also reported in *Streptococcus parasuis* and *Vagococcus lutrae* [50, 51]. Remarkably, the *E. faecium* strain carrying the *poxtA* gene was highly resistant to linezolid (MIC = 32 µg/ml). However, no other resistance genes or 23S rRNA mutations associated with linezolid were detected in this strain. Additionally, this study also identified several *rplC* and *rplD* mutations in enterococci that may be associated with linezolid resistance. Among these, the C348T mutation in *rplD* was most frequently detected in linezolid-resistant *E. faecalis* strains (32.8%, 20/61). The C348T mutation in the *rplD* gene has been reported and may be associated with low-level linezolid resistance in enterococci [24]. In the linezolid-intermediate *E. faecium* strain L1 (MIC = 4 µg/ml), we only identified the *rplC* (A75T, T93G, T495C, C537A, T585G, T600C) and *rplD* (C174T, C180T) mutations. These mutations may be responsible for the elevated level of resistance to linezolid in *E. faecium* strain L1, but further experimental validation is needed.

As *optrA* gene was the most common resistance mechanism identified in the LRE strains in this study, further bioinformatics analyses could be of interest. We found that RDK variant dominated in all variants, but its evolutionary relationship was most distant from the wild-type in the phylogenetic tree. The MIC values of these strains carrying the RKD variant for linezolid were in the range of 8–32 µg/ml, whereas the strains carrying the wild-type *optrA* gene showed the MIC range for linezolid being 8–48 µg/ml. A previous study demonstrated that enterococci strains (isolated from asymptomatic healthy humans) carrying the wild-type *optrA* gene or the RDK variant exhibited relatively high levels of resistance to linezolid compared to other variants [52]. Another study demonstrated by transformation experiments that recipient carrying the RDK variant (MIC = 4 µg/ml) increased the MIC of linezolid relative to the original recipient strain (MIC = 2 µg/ml), but failed to reach the MIC of wild-type *optrA* gene recipient strain (MIC = 8 µg/ml) [9]. Thus, distinct variants of the *optrA* gene may confer differential resistance to linezolid in enterococci. On the other hand, it also highlighted the critical role of *optrA* gene in the resistance mechanism of linezolid. Importantly, we have identified four novel variants (D, KDS, KLDD, and EDS, MIC: 8–24 µg/ml) which have not been reported in the previous study. To fully understand the functions of these new variants, plasmids carrying the above variants can be constructed and transfected into the recipient organisms for further studies in the future.

In previous reports, the *optrA* gene was detected in *E. faecalis* at a higher rate than in *E. faecium* [52]. It has been found that the *optrA* gene can integrate into the genome (plasmids or chromosomes) of enterococci [53, 54], *Vagococcus lutrae* [51], *Clostridium perfringens* [55], *Streptococcus suis* [56], and other species. In this study, we identified that the chromosomal *optrA* was present in the Tn*6674*-like platform, which had been reported in the linezolid-resistant *E. faecalis* isolated from food-producing animals [57] and surface water [58]. Other *optrA*-carrying plasmid arrangements [*impB*-*fexA*-*optrA*, *impB*-*fexA*-*optrA*-*erm*(A), *fexA*-*optrA*-*erm*(A), and *fexA*-*optrA*] identified in this study was also appeared in the linezolid-resistant *E. faecalis* in previous studies [52, 57, 59]. Furthermore, we observed the Tn*6674*-like platform and insertion sequences IS*Vlu1* and IS*1297* around the *optrA* gene. However, in a large-scale study of *optrA*-positive enterococci in Hangzhou, China, Cai et al. indicated that Tn*554*, Tn*558* transposon, and IS*1216E* may be associated with the transmission of the *optrA* gene in enterococci [52]. These results suggest that the transmission mechanism of the *optrA* gene is more complex and not fixed. Through analyzing the upstream and downstream genes, we found that the *optrA* gene was adjacent to multiple resistance genes such as *fexA*, *aadE*, *spc*, and *erm*(A) (Fig. 3). It implies that LRE carrying the *optrA* gene may also have potential phenicol (*fexA*-mediated), streptomycin (*aadE*-mediated), spectinomycin (*spc*-mediated), streptogramin, lincosamide, and macrolide [*erm*(A)-mediated] resistant property, which warrants vigilance.

However, this study still has some shortcomings. Due to read length limitations, whole-genome sequencing of bacteria based on the NGS technique may not generate fined genome maps (chromosome or complete genome), thus preventing in-depth and comprehensive analyses of the location of linezolid resistance genes and transmission mechanism. Furthermore, we identified new OptrA variants and mutation sites in *rplC* and *rplD* genes. But the association between these findings and the level of linezolid resistance needs to be verified by further experiments. In summary, our study suggested that multiple mechanisms of linezolid resistance exist among clinical LRE strains in China. This study also addressed knowledge gaps and provided data support for monitoring linezolid resistance in enterococci.

### Electronic supplementary material

Below is the link to the electronic supplementary material.

Table S1: Clinical data of 67 enterococcal strains

Tables S2: Genome quality assessment results of 67 enterococcal strains

Table S3: Linezolid resistance mechanisms of 67 enterococcal strains

Table S4: Predicted results of amino acid mutation effects

Figure S1: The overall design of this study

Figure S2: The sequence typing of 67 enterococcal strains. (**A**) ST statistics of *E. faecalis*. (**B**) ST statistics of *E. faecium*. The colors in the bar graph represent the departments of strains

## Data Availability

Genomes of 67 enterococcal strains analyzed in this study has been deposited in the NCBI database under accession number PRJNA1008900.
