# Supplementary figures and images for: Genomic epidemiology reveals multiple mechanisms of linezolid resistance in clinical enterococci in China
Source: Ann Clin Microbiol Antimicrob. 2024 May 4;23:41. doi: 10.1186/s12941-024-00689-0 (PMC11070108; doi:10.1186/s12941-024-00689-0)

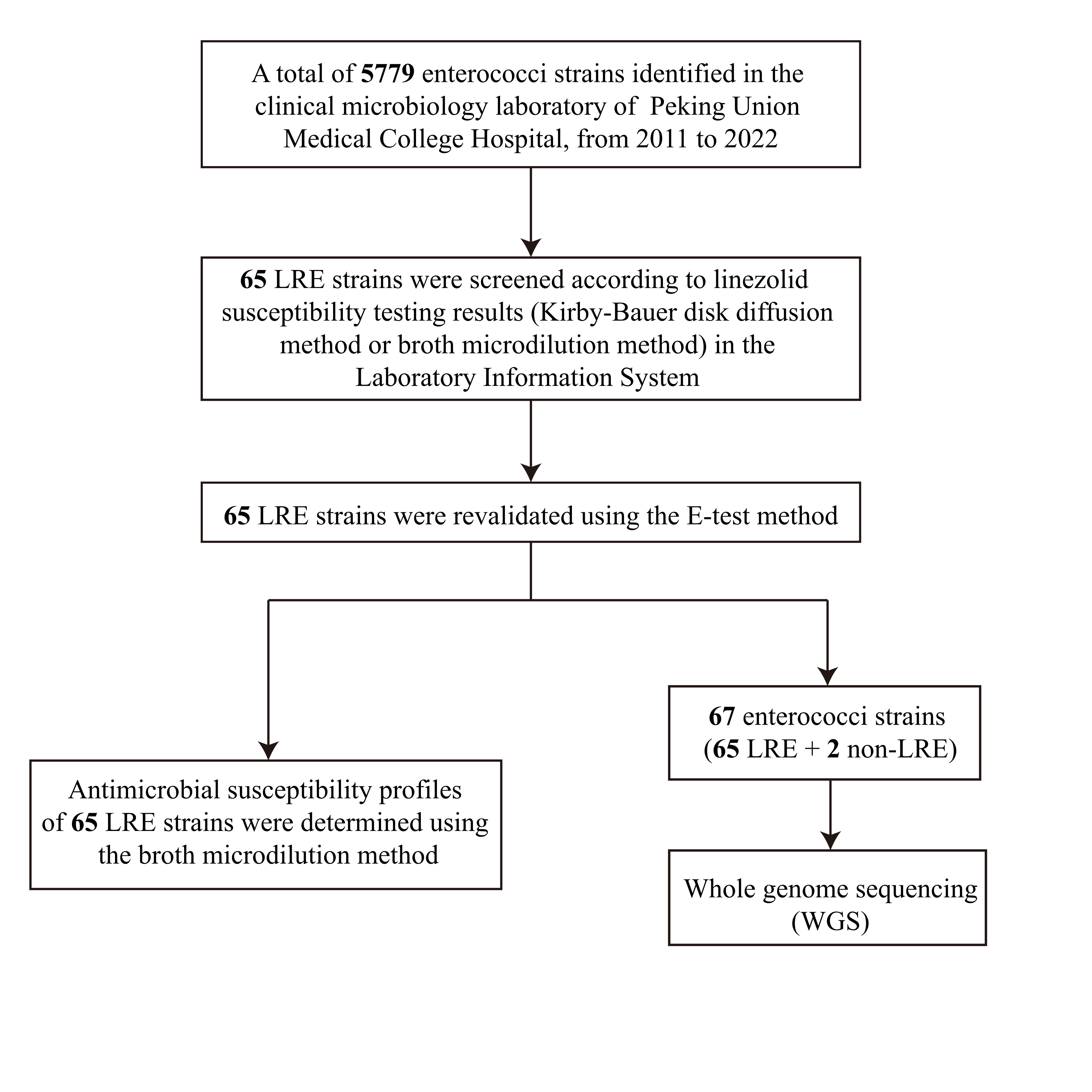

Supplement: Supplementary file 5 — Figure S1: The overall design of this study [file 12941_2024_689_MOESM5_ESM.png]

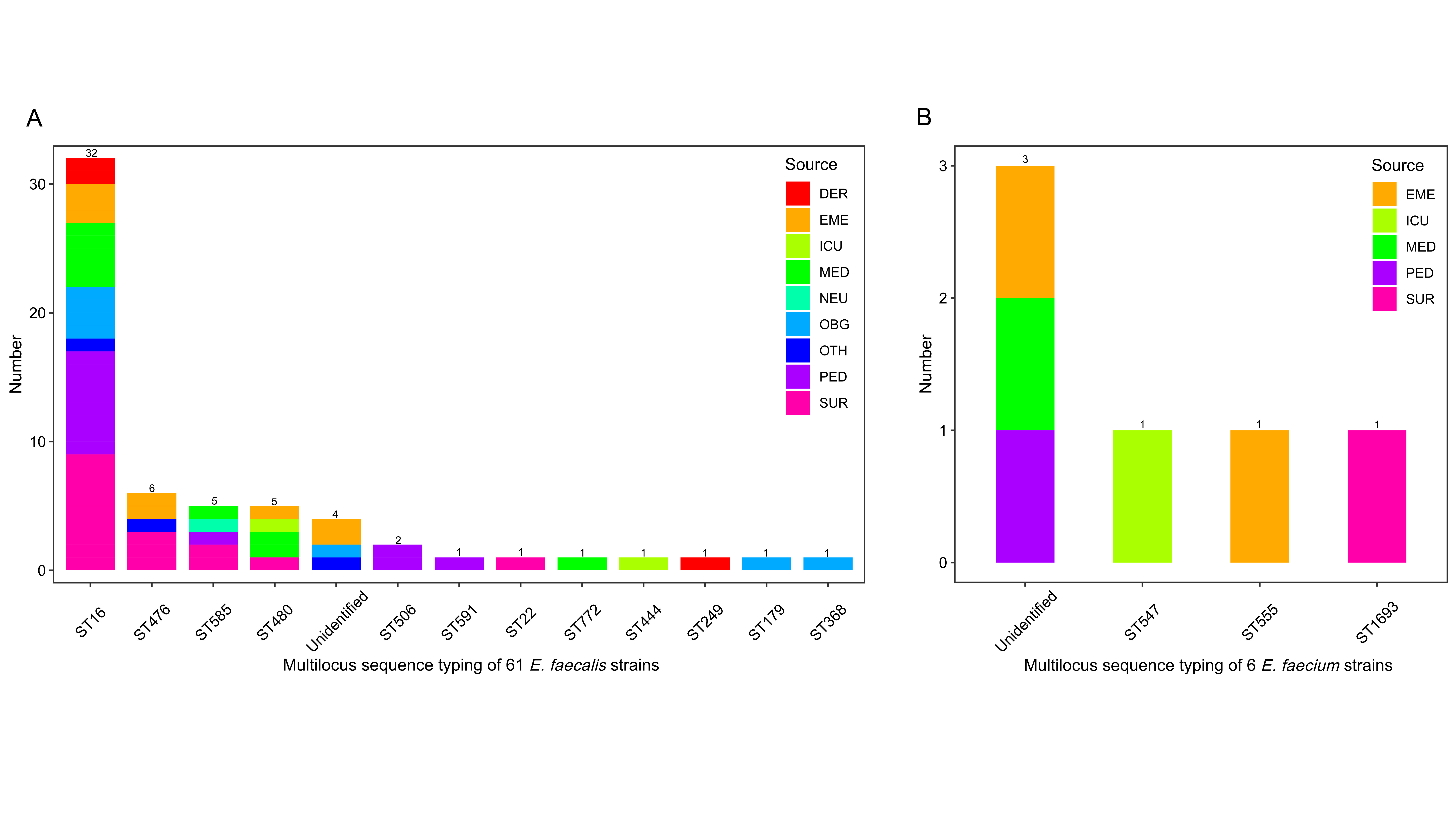

Supplement: Supplementary file 6 — Figure S2: The sequence typing of 67 enterococcal strains. (A) ST statistics of E. faecalis. (B) ST statistics of E. faecium. The colors in the bar graph represent the departments of strains [file 12941_2024_689_MOESM6_ESM.png]
